# Heuristic Optimization of Deep and Shallow Classifiers: An Application for Electroencephalogram Cyclic Alternating Pattern Detection

**DOI:** 10.3390/e24050688

**Published:** 2022-05-13

**Authors:** Fábio Mendonça, Sheikh Shanawaz Mostafa, Diogo Freitas, Fernando Morgado-Dias, Antonio G. Ravelo-García

**Affiliations:** 1 Higher School of Technology and Management, University of Madeira, 9000-082 Funchal, Portugal; 2 Interactive Technologies Institute (ARDITI/ITI/LARSyS), 9020-105 Funchal, Portugal; sheikh.mostafa@tecnico.ulisboa.pt (S.S.M.); diogo.freitas@m-iti.org (D.F.); morgado@uma.pt (F.M.-D.); antonio.ravelo@ulpgc.es (A.G.R.-G.); 3 Faculty of Exact Sciences and Engineering, University of Madeira, 9000-082 Funchal, Portugal; 4 NOVA Laboratory for Computer Science and Informatics, 2829-516 Caparica, Portugal; 5 Institute for Technological Development and Innovation in Communications, Universidad de Las Palmas de Gran Canaria, 35001 Las Palmas de Gran Canaria, Spain

**Keywords:** 1D-CNN, ANN, CAP, HOSA, LSTM

## Abstract

Methodologies for automatic non-rapid eye movement and cyclic alternating pattern analysis were proposed to examine the signal from one electroencephalogram monopolar derivation for the A phase, cyclic alternating pattern cycles, and cyclic alternating pattern rate assessments. A population composed of subjects free of neurological disorders and subjects diagnosed with sleep-disordered breathing was studied. Parallel classifications were performed for non-rapid eye movement and A phase estimations, examining a one-dimension convolutional neural network (fed with the electroencephalogram signal), a long short-term memory (fed with the electroencephalogram signal or with proposed features), and a feed-forward neural network (fed with proposed features), along with a finite state machine for the cyclic alternating pattern cycle scoring. Two hyper-parameter tuning algorithms were developed to optimize the classifiers. The model with long short-term memory fed with proposed features was found to be the best, with accuracy and area under the receiver operating characteristic curve of 83% and 0.88, respectively, for the A phase classification, while for the non-rapid eye movement estimation, the results were 88% and 0.95, respectively. The cyclic alternating pattern cycle classification accuracy was 79% for the same model, while the cyclic alternating pattern rate percentage error was 22%.

## 1. Introduction

Sleep is a complex cyclical process that is usually examined using sleep-related metrics attained from signals recorded by polysomnography (PSG). This examination is considered the gold standard for sleep analysis. The scoring rules, defined by the American Academy of Sleep Medicine (AASM) manuals, assign to each thirty-second epoch (standardized scoring epoch) either the stage wake, Rapid Eye Movement (REM), or one of the Non-REM (NREM) stages [1].

The electroencephalogram (EEG) signals are used as a reference to define the sleep structure, which is composed of macrostructure and microstructure. The macrostructure is a stepwise profile characterized by repetitive NREM and REM cycles, according to the prevalent EEG activity, while transient and phasic events, shown in the brain’s electrical activity, define the microstructure [2]. The paradigm composed of epochs lasting one second was employed to score the microstructure events since they have a shorter duration than the standardized scoring epoch [3].

The Cyclic Alternating Pattern (CAP) concept was proposed by Terzano et al. [4] to examine the microstructure of the NREM sleep by evaluating the sequences of transient electrocortical events, which are different from the EEG background activity. Specifically, the CAP is composed of an activation phase (A phase), characterized by a sequence of transient EEG variations, directly followed by a quiescent phase (B phase), denoting the intermittent recovery of background activity. Each phase can only be considered a valid CAP phase if its duration ranges from two to sixty seconds [4].

Several studies have been carried out to understand the role of CAP in the sleep process. It was proposed that CAP is significantly related to the creation, consolidation, and disruption of the sleep macrostructure [5]. CAP was identified as an EEG marker of sleep instability [6], functioning as a measure of the brain’s effort to preserve sleep [7], thus, working as a sleep quality marker. Temporal relation between behavioral activities, autonomic functions, and CAP was observed [8]. Consequently, CAP was found to be correlated with the occurrence of several disorders, such as sleep apnea [9]. These works advocate the relevance of the CAP as a sleep quality marker. However, a large amount of information is generated during a full night EEG recording. Thus, manual scoring all the CAP events is unpractical, and misclassifications are likely to occur. As a result, the specialist agreement, when analyzing the same EEG signals, ranges from 69% to 78% (getting closer to the lower bound as the number of specialists involved in the analysis increases) [10,11]. Therefore, the development of automatic CAP detection algorithms is desirable and consubstantiates the necessity of this study. The main goal of the developed work is to create an automatic classifier for CAP assessment, which can be used to predict sleep quality.

Each A phase can be divided into three subtypes according to the amplitude and spectral characteristics of the EEG signal [4]. Several works have proposed automatic methods for classifying these subtypes [12,13]. Although these subtypes provide relevant information regarding the sleep process, for the sleep quality examination, the most relevant information is in the occurrence or not of CAP cycles to calculate the CAP rate (total CAP duration to the total NREM sleep duration ratio [4]). This metric is the most widely used microstructural parameter for clinical purposes [8]. It has the advantage of being characterized by a low night-to-night intraindividual variability, thus, allowing the appraisal of the quality of sleep by knowing the subject’s age (a CAP rate higher than the average for the subject’s age can be linked to poorer sleep quality [8]).

Most state-of-the-art works performed the A phase detection by feeding features, created by a feature creation process, to the classification procedure, which is either composed of tuned thresholds or a machine learning classifier. However, the feature creation process requires significant domain-specific knowledge. It is becoming considerably challenging to discover a new set of features that can achieve a higher performance than the methods reported, in the state-of-the-art. It is also relevant to note that combining two or more features does not ensure performance improvement, and the features usually need to be sorted to find the most relevant [14]. These difficulties can be resolved by using a deep learning classifier that automatically learns the relevant patterns directly from the input signal. These classifiers were identified in this work as Automatic Feature Creation (AFC) models.

Nonetheless, important patterns can only be found if there is enough data to train the classifier. Therefore, CAP analysis can become considerably challenging since the classification is based on a second by second evaluation with few data points. For this reason, a novel approach was followed in this work, evaluating consecutive overlapping windows which fed a One-Dimension Convolutional Neural Network (1D-CNN) that can exploit spatially local correlations in the signal by enforcing a local connectivity pattern amongst neurons of adjacent layers. Consequently, the 1D-CNN has the inherent capability to fuse the feature extraction (automatically identifying the distinctive patterns related to the A phases) and the classification processes into a single adaptive learning model. These models are relatively simple to train (compared to the large deep learning classifiers) and have minimal computational complexity, while attaining state-of-the-art performance levels of the complex deep learning models [15]. On the other hand, CAP was found to have temporal dependencies that can be identified by a recurrent neural network [16], such as the Long Short-Term Memory (LSTM). These classifiers were also previously found to be suitable for signal analysis [17]. Therefore, both 1D-CNN and LSTM were examined in this work. 

It was reported by Mendonça et al. [18] that the deep learning models have difficulties recognizing the relevant patterns for two of the three subtypes, which compose the A phases, suggesting the need for examining feature-based methods in this work. Specifically, the LSTM was examined since it was identified as a suitable classifier for feature-based analysis with temporal dependencies [19]. The Feed-Forward Neural Network (FFNN) was also tested as it was identified in the state-of-the-art as possibly the best conventional classifier for A phase estimation, working as a benchmark for the other examined classifiers [20].

As a result, two approaches were followed in this work to perform both the A phase and NREM assessment. The first involved the AFC methodology, where the classifier performed the classification by evaluating the EEG signal without having an explicit feature creation. This work aims to assess if a model based on a machine learning classifier is suitable for CAP and sleep quality assessment and to identify if either AFC or feature-based models are the most appropriate to perform the CAP examination.

The main novelties of this article are:

- Presentation of a novel algorithm for optimizing the structure of deep learning models (code is publicly available). The optimization of deep learning models’ structure is a challenging task. As a result, there is a need for simple algorithms that can allow users to develop new models without requiring a detailed optimization procedure;
- Proposal for a fully automatic sleep stability analysis based on CAP, which provides the A phase, CAP cycle, and CAP rate assessments. To the authors’ best knowledge, this is the first time a single algorithm provides all these metrics with such high accuracy;
- For CAP analysis, the performance of the machine learning models, using features, and deep learning models, with automatic feature extraction, was compared. To the authors’ best knowledge, this is the first time this examination was carried out.

This work has the following organization: evaluation of the state-of-the-art in Section 2; presentation of the materials and methods in Section 3; performance assessment of the developed algorithms in Section 4; discussion of the results in Section 5; conclusions of the work in Section 6.

## 2. State-of-the-Art

Several works have proposed methods for the A phase detection, where the approach of considering each epoch as either “A” or “not-A” is common, leading to a binary classification problem. A technique to describe the sleep microstructure was proposed by Barcaro et al. [21], computing five band descriptors (one descriptor for each of the EEG characteristic bands), which provides a normalized measure of how much the amplitude in a particular frequency band differs from the background. A tuned threshold was then employed to perform the classification. Largo et al. [22] evaluated the signal’s power of five frequency bands (by calculating the fast discrete wavelet transform) and analyzed two moving averages to identify the occurrence of A phases, classified by comparing with a threshold. Niknazar et al. [23] proposed a classification method that performed a similarity analysis between reference windows (from a database) and the windowed signal presented to the algorithm.

Mariani et al. [24] examined the five band descriptors, Hjorth descriptors, and differential variance (of the EEG signal), performing the classification with tuned thresholds. It was observed that differential variance attained the best performance. These features were also evaluated by Mariani et al. [20,25,26], classifying with a Support Vector Machine (SVM) with a Gaussian kernel, an FFNN, and a Linear Discriminant Analysis (LDA), respectively. Other classifiers were tested in the third work, however, LDA attained the highest accuracy. A method based on variable windows was also proposed by Mariani et al. [27], using three discriminant functions (one for each A phase subtype), which were then combined for the final score. Auto-covariance, Shannon entropy, Teager Energy Operator (TEO), and frequency-domain features (chosen by a sequential forward selection method) were evaluated by Mendonça et al. [28], and multiple classifiers were tested. Best results were attained using an FFNN.

A deep learning approach was proposed by Mostafa et al. [29], classifying two-second segments of the EEG signal with a Deeply-Stacked Auto Encoder (DSAE). A similar approach was employed by Mendonça et al. [16], feeding the EEG signal to an LSTM. Hartmann and Baumert [19] have also used an LSTM to perform the classification, fed with entropy-based features, TEO, differential variance, and frequency-based features.

Two approaches were found in the state-of-the-art for the CAP cycle assessment. The first, employed by Mostafa et al. [29], fed the output of the A phase classifier to an FFNN. The second, used by Mendonça et al. [16,28], provided the A phase classification’s output to a Finite State Machine (FSM) to apply the CAP cycle scoring rules [4].

## 3. Materials and Methods

**Figure 1:**
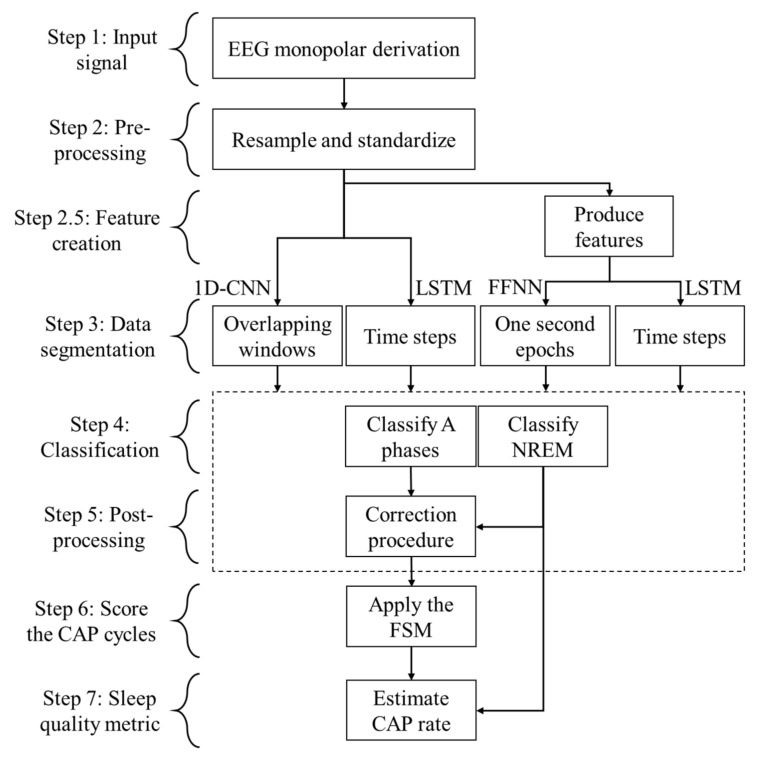
Followed methodology.

AFC and feature-based approaches were developed for the CAP analysis. This was accomplished using the methodology presented in Figure 1 (developed in Python 3 using TensorFlow). The proposed algorithm for the AFC methods is composed of seven steps, starting by pre-processing the input signal, which was then segmented to create either the overlapping windows (for the 1D-CNN) or the time steps data (for LSTM). These were then fed to the classification procedures composed of two parallel classifiers. Each one-second epoch was classified as either “A” or “not-A” by one classifier, and as “NREM” or “not-NREM” by the other classifier. Afterward, a correction procedure was employed, in the post-processing step, to reduce the misclassifications by correcting the isolated “A” or “not-A” classifications and reclassifying the “A” as “not-A” when the NREM classifier indicates a “not-NREM” epoch. The estimation of a sleep quality metric (CAP rate) was performed in the final step. A similar approach was employed for the feature-based methods. However, a new step was included, for the feature creation, between the pre-processing and the data segmentation.

### 3.1. Studied Population

Recordings from nine females and ten males, fifteen free of neurological disorders and four with sleep-disordered breathing, were selected from the Physionet CAP Sleep Database [4,30]. The recordings were performed at the Sleep Disorders Center of the Ospedale Maggiore of Parma. The evaluation was implemented with the EEG monopolar derivation (C3–A2 or C4–A1) signals, which were considered essential for CAP scoring [4]. The relevant characteristics of the population are presented in Table 1. The annotations regarding the sleep macrostructure and the occurrence of the A phases were provided by expert neurologists. The CAP cycles were identified by applying the scoring rules defined by Terzano et al. [4] to the annotated A phases. The total number of examined epochs (each with one second of EEG data) was 592,641.

### 3.2. Pre-Processing Resampling Procedure

The sampling frequency of the records ranges from 100 Hz to 512 Hz. Hence, a resampling procedure was applied to all signals to create a uniform database. Specifically, the records were resampled by decimation [31] at the lowest sampling frequency; therefore, 59,264,100 sample points were examined. A constant reduction factor was used for the sampling rate, *r*, and a standard lowpass filter (Chebyshev type I filter with order eight, a passband ripple of 0.05 dB, and normalized cutoff frequency of 0.8/*r* [32]) was used to down-sample the signal and avoid aliasing. This filter was selected as it is recommended by the standard Python, MATLAB, and R libraries to perform decimation, and also as the Chebyshev type I filters have small transition bands and roll off fast (good properties for decimation). Afterward, the resampling procedure selected each *r*th point from the filtered signal to produce the resampled signal, which was then standardized (subtract the mean and divide the result by the standard deviation) to reduce the effect of systematic signal variations [33]). 

Several studies recommended the removal of artifacts related to movements and the cardiac field to improve the classifier’s performance [13,34]. However, some events can be labeled as an artifact and yet be related to the occurrence of an A phase intended to be detected. The proper removal of eye movement and cardiac field artifacts requires both electrocardiogram and electrooculogram signals, making the algorithm more complex and less suitable for hardware implementation. For these reasons, no artifact removal procedure was employed.

### 3.3. Pre-Processing Segmentation Procedure

A segmentation process was employed to create the epochs. Each epoch corresponds to one label of the dataset, which defines the second (epoch’s duration) as either “A” or “not-A” for the A phase classification, and as either “NREM” or “not-NREM” (not-NREM includes REM and wake periods) for the NREM classification. However, each epoch contains 100 sample points (signal resampled at 100 Hz), which may not be enough for the 1D-CNN classifiers to find the relevant patterns. Therefore, overlapping windows were examined for this classifier to evaluate if additional information can improve the classification’s performance. Three approaches were tested for the overlapping, considering either the first, the central, or the last 100 samples of the window as the ones corresponding to the epoch’s label. Therefore, the first scenario overlaps on the right, the second on the right and left, and the third on the left. 

For the LSTM classifier, the time steps concept was employed where the features fed each LSTM block, and were either from an epoch of the pre-processed input signal or from the features created in the feature creation procedure. The number of time steps and the number of hidden units that produced the LSTM layer’s outputs are parameters that require tuning.

### 3.4. Feature Creation

A feature creation procedure was used for the feature-based methods, and three categories of features were examined. The first category was composed of features produced from symbolic dynamics, performing segmentation analysis, and one amplitude variation metric. These features identify the abrupt variations in the signal’s amplitude that occur during the A phases. The symbolic dynamics transformed the input signal into a sequence of symbols by examining several thresholds for the signal’s amplitude, which are multiples of the signal’s standard deviation, *σ*. A total of nine thresholds were used since it was previously identified as a suitable number for the A phase examination [18]. Thus, for each sample point of the epoch (which is composed of 100 sample points), the algorithm evaluated if the point’s amplitude is lower than either −5 × *σ*, −4 × *σ*, −3 × *σ*, −2 × *σ*, −*σ*, 2 × *σ*, 3 × *σ*, 4 × *σ*, emitting the symbol 1, 2, 3, 4, 5, 6, 7, 8, 9, respectively. The number of each emitted symbol (for each input window) was then considered as the value for the feature. As an example, if symbol 1 was emitted 10 times, symbol 2 was emitted 15 times, symbol 3 was emitted 5 times, symbol 4 was emitted 10 times, symbol 5 was emitted 50, and symbol 6 was emitted 10 times, then the value for the features *A*_1_ to *A*_9_ are 10, 15, 5, 10, 50, 10, 0, 0, 0, respectively.

An amplitude variation metric was also examined by calculating the variation in the current epoch’s, *E*, maximum amplitude with respect to the previous two epoch’s maximum amplitudes by

(1) $$A\left(E\right)=\max\left(E\right)+\left[\max\left(E-2\right)-\max\left(E-1\right)\right]$$

where max is the operation that searches for the maximum value. This feature was used since it can possibly designate the onset epoch of an A phase as, by definition, the amplitude of the epoch must be 2/3 higher than the previous two epochs [4].

The second category of features examined the Power Spectral Density (PSD) of the five characteristic EEG frequency bands, specifically, Delta (*PSD_D_*), Theta (*PSD_T_*), Alpha (*PSD_A_*), Sigma (*PSD_S_*), and Beta (*PSD_B_*). These features were employed as they were previously identified as relevant for A phase analysis as the A phases are composed of characteristic frequency patterns on these bands [28]. The PSD was calculated using the Welch’s method with the Hanning window, *H*, and an overlap, *φ*, of 50% for a given frequency, *ζ*, by [35].

(2) $$\beta \left(\zeta \right)=\frac{\sum_{i=1}^{\mathrm{floor}\left[\frac{P-\varphi M}{\left(1-\varphi \right)M}\right]}{\left|\sum_{n=0}^{M-1}x_{i}\left(n\right)H_{M}\left(n\right)e^{-j2\pi \zeta n}\right|}^{2}}{\mathrm{floor}\left[\frac{P-\varphi M}{\left(1-\varphi \right)M}\right]\sum_{n=0}^{M-1}{\left[H_{M}\left(n\right)\right]}^{2}}$$

where *P* is the number of points in the evaluated segment, *M* is the examined segment’s length, floor is the floor function, and *x* is the input signal.

The last category of features combined the concepts from the two previous categories by calculating the ratio of the maximum (max) amplitude’s value of the epoch to the assessed PSD of the epoch for each evaluated EEG frequency band by

(3) $$\alpha \left(E,\zeta \right)=\frac{\max\left(E\right)}{\beta \left(\zeta \right)}\;$$

denoting as *APSD_D_* for the delta band, *APSD_T_* for the theta band, *APSD_A_* for the alpha band, *APSD_S_* for the sigma band, and *APSD_B_* for the beta band. These ratio based features were considered since they combined the information of both time and frequency, which are relevant for the A phase assessment as the activation phases are composed of phasic and transient activities [4].

The relevance of the features for the A phase classification was assessed by the Minimal-Redundancy-Maximal-Relevance (mRMR) algorithm, which is a classifier-independent method [36]. This algorithm assessed the maximal statistical dependency criterion considering the mutual information *θ*, which for two discrete variables, *I* and *J*, is defined as

(4) $$\left(I,J\right)=\sum_{r,s}P\left(I=i_{r},J=j_{s}\right)\log\left[\frac{P\left(I=i_{r},J=j_{s}\right)}{P\left(I=i_{r}\right)P\left(J=j_{s}\right)}\right]$$

The maximum dependency on the target class, *ρ*, was assessed individually by evaluating the dependence of the selected features *ψ_τ_* (for *τ* = 1, 2, …, *L*) through [36].

(5) $$D\left(L,\rho \right)=\max\left(\frac{\sum_{\psi_{u}\epsilon L}\theta \left(\psi_{u},\rho \right)}{\left|L\right|}\right)$$

The evaluation of the minimum (min) redundancy lessens the issue of large dependency among the selected features and was performed by

(6) $$R\left(L\right)=\min\left(\frac{\sum_{\psi_{u},\psi_{v}\epsilon L}\theta \left(\psi_{u},\psi_{v}\right)}{{\left|L\right|}^{2}}\right)$$

The algorithm ranked the features by simultaneously estimating *D* and *R* through the operation.

(7) $$\mu \left(D,R\right)=\max\left(D-R\right)$$

The features were ordered by the mRMR ranking from most to less relevant. The optimal number of features was identified by testing the 20 possible feature sets, where the first was composed of only the feature identified as the most relevant, the second by the two features identified as the most relevant, and so on, up to the last set, which was composed of all features. The features that composed the set that attained the highest performance for the considered reference performance metric were selected for the performance examination.

### 3.5. Classification

Three machine learning classifiers were tested to perform the A phase and NREM detection. The FFNN is a conventional shallow neural network composed of one input layer, one hidden layer, and one output layer. Each neuron of the network applies an activation function, *Γ*, that considers the bias, *B*, the number of connections, *C*, and their weight, *W*, through [37].

(8) $$Y=\Gamma \left(\sum_{a=1}^{C}x_{a}\times W_{a}\right)+B$$

The hyperbolic tangent function was selected to be the activation function, defined as [37].

(9) $$\tan h\left(x\right)=\frac{2}{\left(1+e^{-2x}\right)}-1$$

The soft-max function was used as the activation function of the output layer to provide a probabilistic score according to the probability distribution *χ* for the input *g* over the *Q* possible results through [37].

(10) $$\mathrm{softmax}\left(\chi^{\left(g\right)}\right)=\frac{e^{\chi^{\left(g\right)}}}{\sum_{q=1}^{Q}e^{\chi^{\left(q\right)}}}$$

For the CNN classifier, the model with one dimension was selected since it can identify relevant patterns from challenging one-dimensional biomedical signals, using a small number of neurons and hidden layers [16,38,39,40]. The small networks are easier to train and implement, requiring less computational resources to develop the algorithm [14].

The 1D-CNN was composed of a sequence of three main groups of layers. First was the input layer, followed by groups of convolution and pooling layers, and classification layers formed the last group. The transformation of the inputs was performed by convolution operations, ⊛, on the convolution layers by [37].

(11) $$c_{n}=\Gamma \left(K_{n}⊛X+B_{n}\right)$$

where *n* is the number of kernels (*K*), and *X* are the inputs. These layers allowed the recognition of the most relevant patterns present on the physiologically driven signal for the desired classification. The Rectified Linear Unit (ReLU) was employed as the activation function that supports these layers’ complex pattern learning since it can provide a good classification performance while diminishing the vanishing gradient problem [37]. The ReLU is defined as [37].

(12) $$\mathrm{ReLU}\left(x\right)=\left\{\begin{array}{c} 0,\;x<0\\ x,x\geq 0 \end{array}\right.$$

The data dimensionality was reduced by employing a subsampling layer after the convolution layer. For this purpose, a max-pooling operation was used, mapping a sub-region to its maximum value. This layer regulates the networks’ complexity and reduces overfitting, which improves the generalization capability [37].

Fully connected (dense) layers were used at the end of the network to improve the learning ability of the nonlinear parameter and perform the classification [37]. Specifically, two dense layers were employed. The first was located between the last subsampling layer and the output layer to map the data (using the ReLU as the activation function). The output layer applied the soft-max function (providing a probabilistic score for each class). 

All memory cells of the LSTM are controlled by three gates at each time step *z*. For the input signal *x_z_*, the input (*i*) and output (*o*) gates control the flow of activations through [41].

(13) $$i_{z}=\Gamma \left(W_{i}x_{z}+\Omega_{i}h_{z-1}+B_{i}\right)$$

(14) $$o_{z}=\Gamma \left(W_{o}x_{z}+\Omega_{o}h_{z-1}+B_{o}\right)$$

where the sigmoid function was used as activation function, defined as [37].

(15) $$\mathrm{sigmoid}\left(x\right)=\frac{1}{1+e^{-x}}$$

*Ω* are the recurrence weights, and *h* is the hidden state given by

(16) $$h_{z}=o_{z}\Gamma \left(s_{z}\right)$$

using the hyperbolic tangent function as the activation function, and *s* is the cell state, defined as

(17) $$s_{z}=f_{z}s_{z-1}+i_{z}\left[\Gamma \left(W_{s}x_{z}+\Omega_{s}h_{z-1}+B_{s}\right)\right]$$

where activation function was the same as Equation (16) and *f* is the forget gate given by

(18) $$f_{z}=\Gamma \left(W_{f}x_{z}+\Omega_{f}h_{z-1}+B_{f}\right)$$

Both *s* and *f* used the sigmoid function as the activation function. A dense layer was employed as the output layer, applying the soft-max function. The output class of all classifiers was given by the highest score through a max operation.

### 3.6. Post-Processing Procedure and CAP Assessment

A correction procedure was applied in the post-processing to correct misclassifications and was composed of two stages. Considering the shortest possible A phase lasts two seconds, and that binary classification provided an output for every second, thus, an output class bounded by two opposite classes (isolated classification) was treated as an error. Hence, in the first stage, a succession of 101 was corrected to 111 and 010 to 000. The NREM classification was then used in the second stage. Taking into consideration that CAP is only defined in the NREM sleep, consequently, if the A phase classifier referenced an epoch as “A” when the NREM classifier indicated as “not-NREM”, then the “A” was reclassified as “not-A”.

The correction procedure’s outputs fed an FSM, which implements the CAP scoring rules [4] to assess the CAP cycles. CAP rate was the estimated sleep quality metric and it was calculated by dividing the total number of epochs classified as CAP (output of the FSM) by the total number of epochs classified as NREM.

### 3.7. Performance Assessment and Optimization of the Classifiers

The performance of the developed algorithms was measured by considering the Accuracy (Acc), Sensitivity (Sen), and Specificity (Spe), defined as [42].

(19) $$\mathrm{Acc}\;=\frac{t_{p}+t_{n}}{t_{p}+t_{n}+f_{p}+f_{n}}$$

(20) $$\mathrm{Sen}\;=\frac{t_{p}}{t_{p}+f_{n}}$$

(21) $$\mathrm{Spe}\;=\frac{t_{n}}{t_{n}+f_{p}}$$

where *t_p_*, *t_n_*, *f_p_*, and *f_n_* are the true positives (for the A phase assessment, it reflects the number of epochs related to an activation phase correctly identified, while for the NREM classification, it indicates the number of epochs related to the NREM periods correctly recognized), true negatives (for the A phase classification it indicates the number of epochs related to the “not-A” class correctly recognized, while for the NREM assessment it indicates the number of epochs related to the “not-NREM” class correctly identified), false positives, and false negatives, respectively. The diagnostic aptitude of the classifiers was assessed by the Area Under the receiver operating characteristic Curve (AUC) [43]. The Significance of the results was determined according to the Wilcoxon rank sum test (left-tailed), displaying the *p*-value when comparing the results against the FFNN (standard model used as a benchmark), evaluating how significant performance improvements are. The statistical analysis was performed considering a significance level of 0.05.

The FSM performed the CAP cycles classification, hence, no probabilistic output was created, and the AUC was not computed. However, the CAP rate error and the CAP rate percentage error were assessed as predictive metrics of the overall capability of the model to estimate the CAP rate, and these metrics were calculated by

(22) $$\mathrm{CAP}\;\mathrm{rate}\;\mathrm{error}\;=CAP_{P}-CAP_{a}$$

(23) $$\mathrm{CAP}\;\mathrm{rate}\;\mathrm{percentage}\;\mathrm{error}\;=\frac{\mathrm{abs}\left(\mathrm{CAP}\;\mathrm{rate}\;\mathrm{error}\right)}{CAP_{a}}\times 100\%$$

where *CAP_P_* is the CAP rate predicted by the developed method, *CAP_a_* is CAP rate assessed by the database labels, and abs is the absolute value function.

The classifiers’ hyper-parameters optimization was empirically performed by a search methodology, selecting the configuration which attained the highest AUC (considered reference performance metric). Random Sub-sampling Validation (RSV) was employed for the optimization procedure, randomly choosing ten subjects to compose the training set and nine for the validation set, ensuring subject independence of the sets. Each validation procedure was repeated ten times to achieve statistically significant results. Error optimization for all classifiers was performed by the Adam algorithm [44] (learning rate of 0.001 and batch size of 1024) to allow a fair comparison of the results. An early stopping procedure was used to reduce the simulation time and avoid overfitting the classifier. The training procedure was stopped (before the end of the maximum number of training cycles, defined as 50) if no relevant improvement in the AUC (improvement lower than 1%) of the validation set was reached within five consecutive epochs. 

A complete grid search optimization approach for all hyper-parameters of the classifiers is not computationally feasible. Therefore, only the most relevant parameters were tuned for each classifier. For the FFNN optimization, the number of neurons employed for the hidden layer was varied from 100 to 400, in steps of 100. On the other hand, the Heuristic Oriented Search Algorithm (HOSA) employed in this work follows the concepts presented by Mendonça et al. [40] and Mostafa et al. [45], for the LSTM or 1D-CNN optimization to assess the most relevant architecture for the classifiers by considering a heuristic search for the parameters considered to be the most relevant for the examined models.

Yamashita et al. [46] identified the most important hyper-parameters to tune a 1D-CNN, where the dominant parameters are the kernel size, number of kernels, and the number of layers [47]. Hence, the performed search concentrated on these hyper-parameters. Considering that the kernel size will define the extent of the features that will be identified and that each sample point of the segmented windows has relevant information, thus, a kernel size of two with a unitary stride was chosen. The optimal number of kernels was identified by starting with a value of eight, which was successively increased by a factor of two without changing the remaining parameters [48]. 

The overlapping duration, *O*, of the segmented windows was iteratively changed (testing the three scenarios of overlapping, *A_p_*, where the database label corresponds to either the first, central, or last second of data from the overlapping window *W*) for each tested combination of the relevant hyper-parameters. The algorithm started without overlapping, and the duration of overlapping was increased in steps of four seconds up to a maximum window, *O_max_*, of 35 s (the upper limit was empirically found to be above the saturation point for the best A phase AUC). The searching procedure was improved by using the group of layers concept (*GofLayer*) [40], where each group was composed of one convolution layer, followed by one subsampling layer, and a 10% dropout was applied at the output of the group. A downsample of factor two was applied in the subsampling layers with the chosen stride and filter size of two. These values are frequently used for 1D-CNN as they can reduce the dimensionality of the data while maintaining the highest excitations from the convolutional feature maps [47]. The employed algorithm for optimization is presented in Table 2 and starts with a network composed of: one input layer (*I_pt_*) where the input data (named *Data*) was fed; one group of layers; two dense output layers (*D_e_*). The number of *GofLayer*, *G*, was iteratively incremented until the maximum value *G_max_* (chosen to be four). The number of kernels *K* of the convolution layer, for the first *GofLayer*, was 16, and the maximum limit was 128, using a step 2*^M^* where *M_start_* ≤ *M* ≤ *M_max_* (*M_start_* and *M_max_* were four and seven, respectively). 

The subsequent *GofLayer* were introduced, with either the same or twice (increment of the multiplier, *MUL_max_*, of two) the number of kernels of the previous group of layers (leading to linear growth in the number of simulations). The value for the number of neurons of the first dense layer (*D_e_*), *N*, started at 50 (*N_start_*), and was incremented in steps of 50 (*N_step_*) until the maximum value of 150 (*N_max_*) was reached. This recurrent process occurred until no relevant improvement in the AUC (considering the minimum threshold, *t_r_*, increase of 1%) was attained, signifying that the best network, *Net*, was found. For the LSTM-based classifier, the input layer, *I_p_*, was followed by either a Bidirectional LSTM (BLSTM) or an LSTM layer. The subsequent evaluated layers were chosen to be equal to the first recurrent layer, and the last recurrent layer of the tested architecture could be followed by a dense layer. The number of recurrent layers, *Gr*, was increased one by one until reaching five, the chosen maximum number (*Gr_max_*), or was stopped earlier if no significant improvement in the AUC was attained (examining a minimum threshold increase of 1%) when comparing with the model with *Gr*–1 layers. The number of time steps, *T*, employed by the recurrent layers was varied from five (*T_start_*) to 35 (*T_max_*) in steps of ten (*T_step_*).

The number of hidden units, *Nh*, used for the recurrent layers (the same *Nh* was used for all recurrent layers for the models with a cascade of LSTM layers) was varied from 100 (*Nh*start) to 400 (*Nh_max_*) in steps of 100 (*Nh_step_*). The *D_e_* weights were initialized with a normal distribution and the number of hidden units was chosen to be either half (applying the *floor* function to round the result), the same, or twice the number of hidden units that were employed by the previous recurrent layer. It was empirically observed that the use of more than 35 time steps or more than 400 hidden units did not lead to a significant increase in performance. A 10% dropout was employed between the last recurrent layer and the first dense layer to reduce the possibility of overfitting. Table 2 presents the algorithm’s pseudo-code.

The NREM classifier was only tuned for the best overlapping scenario (for the 1D-CNN) or best number of time steps (for the LSTM) identified for the A phase classifier since the NREM classifier was fed with the same input as the A phase classification, and is only intended to be used in the correction procedure and for the sleep quality metric estimation. No balancing operation (oversampling the minority class or undersampling the majority class so that all classes have the same number of samples) was implemented in any training or testing dataset since it can alter the expected distribution of the data. Conversely, it was observed that the classifier’s performance could be significantly improved by using cost-sensitive learning (applying a greater cost when misclassifying an element of the minority class compared to an event from the majority class). This observation is particularly relevant for the A phase classification since it is sturdily unbalanced (significantly more “not-A” than “A” events). Hence, this approach was used to develop the classifiers [49].

The performance of the algorithms (whose classifier’s hyper-parameters were previously selected) was evaluated by the Leave One Out (LOO) method as it can provide less biased results for classifiers with few samples [50]. A total of 19 evaluation cycles were performed, each repeated 50 times to attain statistically significant results, considering the average of the performance metrics of the repetitions as the result of the evaluation cycle. For each cycle, the testing dataset was composed of data from one subject (each subject was only once selected to create the testing dataset). The data from the remaining subjects were used to compose the training dataset, hence ensuring subject independent results.

## 4. Experimental Evaluation

Three main examination steps were performed for the experimental procedure. The first and second comprised the development of the AFC and feature-based classifiers, respectively, while the third evaluated the performance of the models for the A phase, NREM, CAP cycle, and sleep quality metric estimations.

### 4.1. Development of the AFC Classifiers

For the 1D-CNN, the number of examined combinations was 2136. Each network was simulated ten times. Thus, the total number of examined classifiers (using RSV) was 21,360. The simulation time required for optimization was significantly reduced by using HOSA when compared with an extensive grid search, which would have required testing all possible combinations of parameters for each classifier while attaining a classifier with good performance. An extensive grid search analysis (exhaustive search) would have resulted in an unreasonable number of simulations as the total number of possible combinations can easily lead to millions of network structures being tested, which would not be computationally viable, and most likely would not considerably improve the performance compared to the attained classifier (using the proposed methodology). The developed algorithm can optimize a network with the size of the 1D-CNN employed in this work, in one to two weeks, depending on the complexity and the number of parameters to be tested. These results are considerably fast, even when compared to other heuristic-based optimization algorithms, such as genetic algorithms, that can require multiple months to finish the simulations (for a network of similar size to the 1D-CNN examined in this work) [14].

The optimal 1D-CNN structure for the A phase classification (identified by the HOSA) was composed of 64 kernels in the first convolution layer, 128 kernels in the second convolution layer, and 100 weights in the first dense layer. For the NREM classification, the classifier was composed of 32 kernels in the first convolution layer and 64 kernels in the second convolution layer, using the same number of weights in the first dense layer. Therefore, it was concluded that the best performance was attained using two groups of layers (*GofLayer*). A similar result was previously reported by Mostafa et al. [45], where it was observed that the use of two clustered layers (composed of one convolution layer, followed by batch normalization and a pooling layer) led to the highest improvement in the considered performance metric.

It was observed that the second scenario for overlapping (epoch’s label refers to the central 100 sample points) attained the best AUC, which was considerably better than the other two scenarios, with the optimal window length of 19 s. This result is likely linked to the average A phase duration, found to be around 13 s [4]; hence, extending the window length too much can possibly introduce excessive information from the background activity, leading to misclassifications.

These results suggest that there is a strong temporal dependency for the A phases as introducing more information to the classifier significantly improved the classification capability. The low performance of the first scenario was associated with misclassifications of the onset boundary. Such a scenario occurred when the current epoch (data points related to the label) was “not-A” and the following epochs were “A” (and the sampling points of these epochs were present on the segmented window), leading the classifier to classify the current “not-A” as “A”. This effect was lessened in the third scenario even though the converse effect occurred, related to the A phase offset boundary detection when the current epoch under classification was “A”, and the sampling points associated with a subsequent “not-A” epochs were present in the segmented window. Consequently, it led the classifier to wrongly classify the current epoch as “not-A”. 

Both onset and offset misclassification issues occurred in the second scenario. However, these were diminished as the classifier has contextual information from the previous and next epochs. It was also observed, for all scenarios, that the proper detection of the offset boundary was challenging, occurring several misclassifications towards the end of the longer A phases where the classifier oscillated between “A” and “not-A”. This effect was previously reported by Terzano et al. [4], indicating that the A phases can display ambiguous limits due to inconsistent voltage changes in the EEG signal. Nonetheless, post-processing lessened this problem (if two consecutive A phases are separated by an interval shorter than two seconds, then they should be combined in a single A phase). However, these oscillations were still the most notable reason for the misclassifications. It was also observed that increasing the window length beyond 31 s (having 30 s of overlapping) was counterproductive as further information led to misclassifications.

For the LSTM-based classifier, it was noticed that the best structure found by the HOSA was composed of an LSTM layer with 100 hidden units using 25 time steps, followed by a dense layer with 50 hidden units. The cascade LSTM architecture led to a lower AUC, and the use of BLSTM instead of LSTM in the recurrent layer had an AUC increase of less than 1%. Therefore, the LSTM was preferred rather than BLSTM since it attained a better complexity to performance ratio. A total of 256 network architectures were examined, and each test was repeated ten times using RSV. Therefore, the total number of evaluated classifiers was 2560. It was observed that the proper offset detection was again the primary source of misclassifications, although the increase in the number of time steps allowed the model to lessen this problem. However, the use of more than 25 time steps led to a lower AUC, possibly suggesting that the model could not extract more relevant information from the input data and started to overfit. The best network’s architecture for the NREM classification using the 25 time steps was composed of one LSTM layer followed by one dense layer, with 300 and 150 hidden units, respectively. It was observed that the best performance was reached when using only one recurrent layer and these results agree with the findings reported by Yadav et al. [51], which have observed that a model with one LSTM layer outperformed models with cascade recurrent layers. 

**Figure 2:**
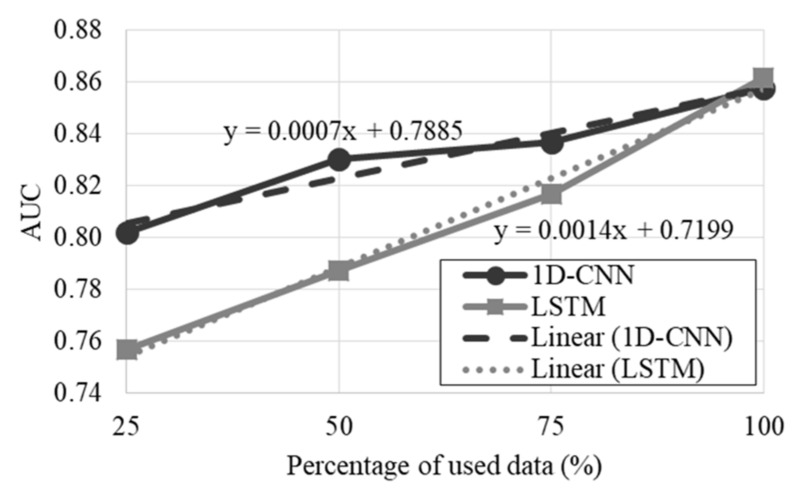
Learning curves of the optimized AFC classifiers.

The learning curves of the classifiers are presented in Figure 2. It was observed that both classifiers could possibly improve the performance if more data were available in the database, and LSTM would perhaps benefit more from the additional data (the slope of the LSTM linear tendency line is higher than the 1D-CNN linear tendency line). On the other hand, the performance of both classifiers is similar when 100% of the data was used for the model’s development, thus, the performed comparative analysis regarding which classifier is more suitable for the intended classification is fairer.

### 4.2. Development of the Feature-Based Classifiers

The relevance of the features for the A phase classification was assessed by the mRMR algorithm (each simulation of the presented results was repeated 50 times to attain statically significant results), and the ordered sequence (from most to less relevant) was: *PSD_D_*; *A*; *PSD_S_*; *PSD_T_*; *APSD_B_*; *PSD_A_*; *APSD_D_*; *APSD_S_*; *APSD_A_*; *PSD_B_*; *APSD_T_*; *A*_3_; *A*_7_; *A*_2_; *A*_9_; *A*_8_; *A*_4_; *A*_1_; *A*_6_; *A*_5_. The *PSD_D_* and *A* features were expected to be the most relevant since 61% of the database’s A phases belong to the A1 subtype that is characterized by high-voltage slow waves, where delta waves are the most prevalent. On the other hand, the A2 subtypes compose 21% of the database labels and have a mixture of high-voltage slow waves with low-amplitude fast rhythms, whereas the A3 subtypes have a predominance of low-amplitude fast rhythms [4]. Therefore, it was anticipated that the frequency-based features would be more relevant for the A phase assessment. However, the amplitude-based features are still important to detect the high-voltage waves.

For the FFNN optimization, each tested value for the number of hidden units was examined for all 20 feature sets ordered by the mRMR algorithm. It was observed that the best performance was attained using the 14 most relevant features with 400 hidden units for both A phase and NREM classifications (using RSV for the performance assessment, repeating each simulation ten times). The structure of the LSTM-based classifiers previously identified as the best for the A phase or NREM classification was employed for the feature-based classification to allow a fairer comparison of the results, and the best performance was attained using the 12 most relevant features. 

**Figure 3:**
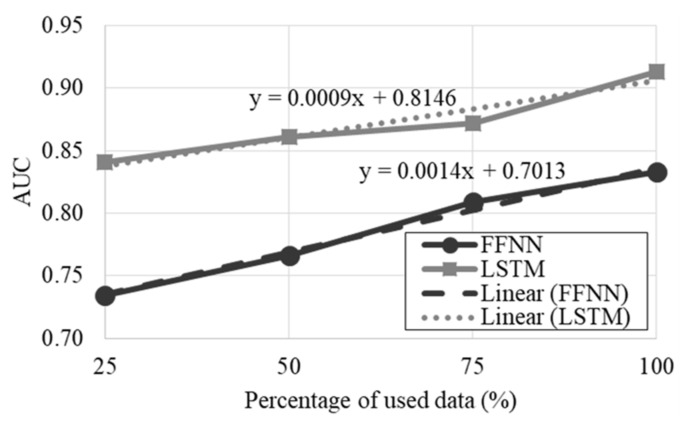
Learning curves of the optimized feature-based classifiers.

The learning curves are depicted in Figure 3. Similar to the AFC models, the inclusion of more data could possibly improve the performance of the classifiers. However, the variation in performance of the LSTM is likely to be significantly lower for the feature-based methods. It was also observed that the LSTM has a significantly higher AUC than the FFNN, suggesting that the performance of the LSTM-based classifier is expected to be superior.

### 4.3. Performance Evaluation

The performance of the tuned classifiers was assessed using LOO, repeating each simulation 50 times to provide reliable estimates of the models’ performance, being the results presented in Table 3 (the Appendix A tables present the results for all subjects, where subjects 1–15 are free of neurological disorders, while subjects 16–19 were diagnosed with sleep-disordered breathing). Regarding the A phase estimation, by examining the table’s results, it is possible to conclude that the FFNN-based classifier attained the lowest Acc, Spe, and AUC while the feature-based LSTM reached the best performance for all performance metrics, having significant improvements when comparing against the FFNN in eight of the eleven studied metrics.

On the other hand, the AFC LSTM attained the most unbalanced results (largest difference between Sen and Spe), suggesting that the AFC classifier could not find patterns in the data that are as relevant as the ones present in the used features. The AFC classifier based on the 1D-CNN surpassed the AFC classifier based on the LSTM for the A phase assessment. However, the opposite occurred in the NREM classification, where the AFC classifier based on the LSTM performed better. The FFNN was the worst classifier for the NREM assessment, while the feature-based LSTM was the best. For the CAP assessment, it was observed that the model which used the AFC classifier based on LSTM attained a better Acc and Sen than the classification based on the 1D-CNN, which reached the highest Spe of all models. The FFNN-based model had the lowest Acc and Sen. 

**Figure 4:**
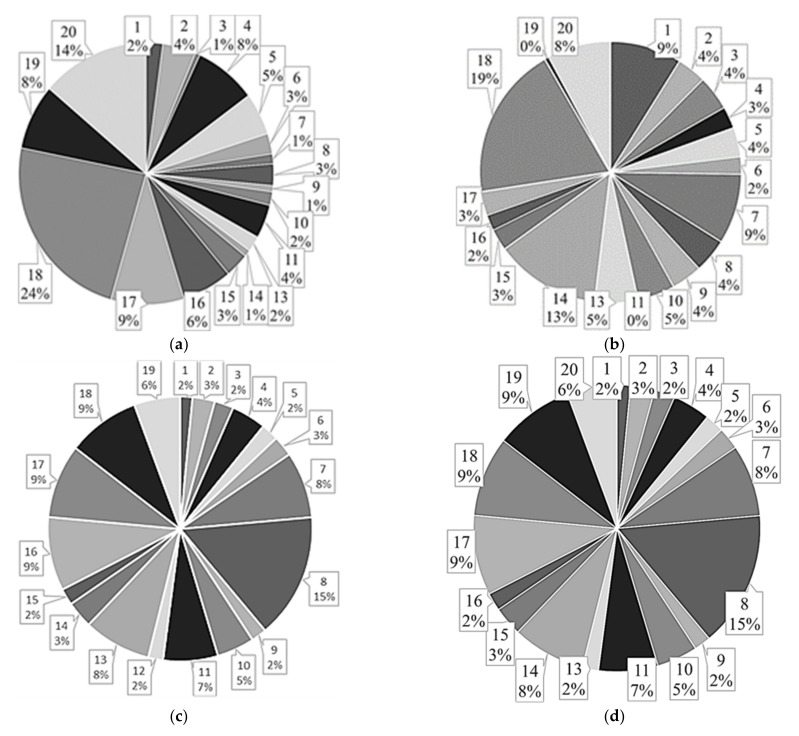
Normalized CAP rate error, for all examined subjects, for the model based on: (**a**) the 1D-CNN; (**b**) the AFC LSTM; (**c**) the FFNN; (**d**) the LSTM fed with features. The subject’s number is presented on a balloon, on the top, followed by the percentage of normalized CAP rate error for the respective subject.

**Figure 5:**
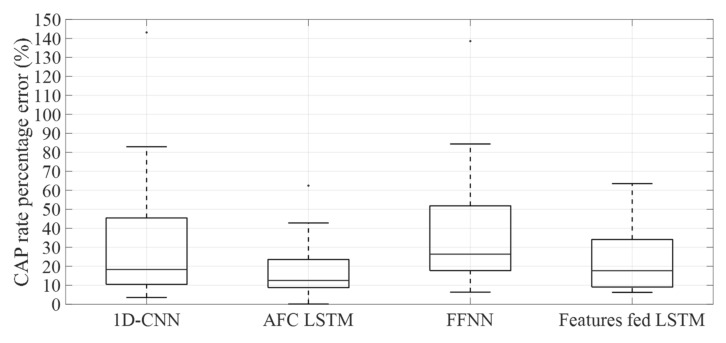
Boxplots of the CAP rate percentage error for all examined classifiers, which performed the A Phase and NREM classifications.

It was observed that the lowest CAP rate percentage error was attained by the model based on the AFC LSTM, while the FFNN-based model had the worst performance. On the other hand, the 1D-CNN and the model with the LSTM fed with features reached a similar average value, although the 1D-CNN results have a larger variation. Figure 4 and Figure 5 depict the normalized CAP rate error and the CAP rate percentage error boxplots, respectively.

It is possible to forecast the sleep quality by knowing the subject’s predicted CAP rate, considering that a higher CAP rate most likely designates a poor sleep quality. In contrast, the reverse probably means good sleep quality [8]. If the subject’s age is known, then sleep quality guess can conceivably be performed by comparing the predicted CAP rate against what is the average CAP rate for the subject’s age, considering that a higher value denotes poor sleep quality and a lower value designates good sleep quality. By following this simplistic approximation, the accuracy of the sleep quality prediction (by comparing with the estimate based on the CAP rate from the dataset) for the 1D-CNN, AFC LSTM, FNN, and feature-based LSTM was 74%, 79%, 68%, and 90%, respectively.

## 5. Discussion

By evaluating the attained results, it is possible to conclude that the use of features leads to the best performance. However, if more data were available, probably, the AFC classifiers would significantly improve the results (as it is visible in Figure 2). A similar conclusion can be attained for the CAP cycle’s assessment. The achieved results are emphasized by the difficulties associated with CAP analysis, as reported by Mendez et al. [12], which predicted that the CAP phase assessment could be affected by up to 25% of subjectivity and ambiguity. Another relevant factor is the specialist agreement for CAP analysis, examining the same EEG signals, which ranges from 69% to 78% (getting closer to the lower bound as the number of specialists involved in the analysis increases) [10,11]. Hence the performance of the proposed algorithms is either in the agreement range or slightly superior to the upper bound, advocating the viability of the algorithms for clinical applications.

The Acc of the CAP cycles classification was lower than the A phase classification, and it was verified that this was due to three factors. The first was the misclassification around the A phase’s offset boundary (oscillation between “A” and “not-A” at the end of the longer A phases), which led the FSM to either overestimate or underestimate the CAP cycles. The second factor was the occurrence of several A phase misclassifications during long “not-A” periods. These mainly occurred during periods of significant variation in the EEG signal, usually lasting more than three seconds and are separated by less than 60 s, leading the FSM to classify these events as a CAP cycle. This second problem was sturdier in the AFC classifiers, possibly suggesting the low Sen. The last factor was the high impact that the NREM classification had in the CAP assessment, where the lower performance created several *f_p_*, and *f_n_*, which led the FSM to either overestimate or underestimate the CAP cycle duration and also affected the CAP rate estimation. It was also observed that the subjects suffering from sleep-disordered breathing were the most challenging to be assessed, conceivably due to the low number of subjects present in the database (when compared with the number of subjects free of neurological disorders) and due to the dynamics of the EEG signal, which are likely to be different for these subjects (with possible variation in the prevalence of each A phase subtype). 

A summary of the results reported by the state-of-the-art, which had performed binary A phase classification is presented in Table 4. Most of the works, which attained a similar accuracy to the proposed work, examined a significantly smaller population for the development of the models. Specifically, Largo et al. [22] tested 12 subjects, considering one hour of data for each subject, Niknazar et al. [23] examined six subjects, Mariani et al. [20,25] evaluated four subjects, and Mariani et al. [26] studied eight subjects. Hartmann and Baumert [19] examined 15 subjects and reached a similar performance as the best model examined in this work. Mariani et al. [27] attained a higher Acc while using a similar population, but with a significantly lower Sen. However, both subjects free of neurological disorders and subjects suffering from sleep-disordered breathing were considered in this work, while the other state-of-the-art results with similar performance have only considered subjects free of neurological disorders. 

It is also relevant to notice that CAP analysis is characterized by a strong unbalance between the number of “A” and “not-A” events (approximately 90% of the database annotations refer to “not-A” events) [18]. Hence a variation in the Spe has a greater impact in the Acc than a variation in the Sen. As a result, a model with a high Spe and low Sen will have a high Acc. This effect can be understood by examining the average metric, which was around 81% for all the best performance works, suggesting that the focus should be on attaining balance results to improve the clinical applicability. Furthermore, even though the traditional methods based on thresholds can achieve a considerable performance with low complexity algorithms, the studies that have examined these methods usually consider a low number of subjects and frequently evaluate only a part of the full-nigh EEG signal. These methods will likely be problematic to generalize to a broader population since the thresholds need to be tuned for the examined population.

A comparative analysis was not implemented for the developed NREM classifiers since no other work was found performing a second by second NREM assessment (the standard defined by the AASM is to use an epoch of 30 s). Nonetheless, the accuracy reported by state-of-the-art works for NREM classification, considering a 30 s epoch, ranges from 72% to 98%, depending on the number of classes considered [52]. Hence the developed work is within the range while using a challenging approach of classifying every second. 

A total of three works were found in the state-of-the-art performing the CAP cycles assessment. Mostafa et al. [29] applied an FFNN for the classification, reporting an Acc of 62%, while Mendonça et al. [16,28] employed an FSM and reported an Acc of 79%, using a feature-based method for the A phase classification, and 76%, when an LSTM classified the A phase. By comparing the results attained in this work, it was concluded that Mendonça et al. [28] reached the same Acc as the developed feature-based LSTM method, while the other works reported a lower performance. However, it is important to bear in mind the higher number of subjects examined in this work. When comparing the AFC-based classifiers, the developed method based on LSTM reached a higher performance for the CAP cycle assessment than Mendonça et al. [16]. 

By examining the normalized CAP rate error presented in Figure 4 it is possible to conclude that subject 17 (subject with sleep-disordered breathing) has the larger normalized error for the models based on 1D-CNN, AFC LSTM, and FFNN, possibly due to the low CAP accuracy of the models for this subject. For the model based on LSTM fed with features, subject 8 was the most challenging, leading to the higher CAP rate error, possibly due to the low A phase accuracy, which led the FSM to overestimate the CAP cycles duration. By examining the CAP rate percentage error, it was observed that the model based on the AFC LSTM has the best results (lowest average value), followed by the model based on LSTM fed with features. The FFNN-based model has the highest average value. By inspecting the boxplots of the CAP rate percentage error presented in Figure 5 it is notorious that the model based on the AFC LSTM has the lowest variation in the results, suggesting that this model is the most suitable for the CAP rate examination. These results are likely to be related to the performance for the CAP assessment since the model based on the AFC LSTM has the most balanced results, with an accuracy that is similar to the best results attained by the model based on LSTM fed with features. 

Only the work reported by Mariani et al. [27] was found in the state-of-the-art performing the CAP rate appraisal. The reported CAP rate percentage error was 17%. The same value was attained by using the model based on the AFC LSTM. Nevertheless, Mariani et al. [27] evaluated only subjects free of neurological disorders, while in this work, subjects diagnosed with sleep-disordered breathing were also examined. Thus, there is a larger variation in the dataset’s CAP rate (most sleep-disordered breathing subjects have a higher CAP rate).

## 6. Conclusions

Two approaches for automatic CAP analysis were developed, estimating the occurrence of the A phases, the CAP cycles, and the CAP rate. The first was based on AFC, where the classifiers automatically identify the relevant patterns from the input data, while the second comprised the use of features created by a feature creation procedure that extract relevant information from the input data to feed the classifiers. It was observed that the feature-based LSTM attained the best performance, although the results for the A phase assessment reached by the 1D-CNN were similar. The performance for the CAP cycle assessment achieved by the feature-based LSTM and the AFC LSTM was similar. These results suggest that the low Sen of the AFC LSTM for the A phase estimation (related to the overestimation and underestimation of the A phase duration) has not affected the CAP cycle assessment. It is also likely that the inclusion of more data could improve the AFC models’ performance, possibly surpassing the feature-based LSTM results. 

The proposed methods perform the analysis by evaluating the signal from only one EEG monopolar derivation without requiring any manual manipulation of the signal or the removal of artifacts. It was observed that the A phase classification performance was similar to the best state-of-the-art algorithms. A second by second based NREM classification was also proposed, which was used in the correction procedure and for the sleep quality metric estimation. The CAP rate error was found to be low, supporting the diagnostic capability of the algorithms for sleep quality estimation. It is important to highlight that the attained results are considerably good when considering the challenges of the bioengineering fields, as the results have even surpassed the specialist agreement when analyzing the same EEG signals, advocating the relevance of the work.

The next steps in this research are to further validate the developed algorithm in a larger dataset and examine the A phase subtypes to reach a deeper understanding of the CAP events, which can lead to a reduction in misclassifications.

## Figures and Tables

**Table 1:** Characteristics of the studied population.

Measure	Mean	Range
Age (years)	40.58	23–78
REM time (seconds)	5652.63	480–11,430
NREM time (seconds)	20,505.79	13,260–27,180
A phase time (seconds)	4059.21	1911–10,554
CAP cycles time (seconds)	10,323.95	5000–23,306
CAP rate (%)	49.16	29–86

**Table 2:** Implementation of the HOSA for 1D-NN and LSTM.

*HOSA-1D-CNN (Data, G_max_, M_max_, M_start_, MUL_max_, N_max_, N_start_, N_step_, O_max_, t_r_)* *G = [1, 2, …, G_max_]* *O = [0, 1, 3, 5, …, O_max_]* *K = 2^M^ where M_start_ ≤ M ≤ M_max_* *N = [N_start_, N_start_ + N_step_, …, N_max_]* *for g = 1 to length (G)* | *for o = 1 to length (O)* | | *for k = 1 to length (K)* | | | *for n = 1 to length (N)* | | | | *if O (o) > 0* | | | | | *W = [2 × O (1) + 1, 2 × O (2) + 1, …, 2 × O (length (O)) + 1]* | | | | | *A_p_ = [W (1), W (floor (W/2 + 1)), W (length (W))]* | | | | *else* | | | | | *A_p_ = 1* | | | | *for a = 1 to length (A_p_)* | | | | | *Net* ← *I_pt_ (Data, O (o), A_p_ (a))* | | | | | *for z = 1 to g* | | | | | *if z == 1* | | | | | | *mul = 1* | | | | | | *Net_g,o,k,n,a,z,mul:MULmax_* ← *Net + GL (K (k))* | | | | | | *k_z,mul:MULmax_ = K (k)* | | | | | *else* | | | | | | *for mul = 1 to MULmax* | | | | | | | *k_z,mul_ = mul × k_z-1,mul_* | | | | | | | *Net_g,o,k,n,a,z,mul_* ← *Net_g,o,k,n,a,z-1,mul_ + GL (k_z,mul_)* | | | | *Net_g,o,k,n,a,z,mul_* ← *Net _g,o,k,n,a,z,mul_ + D_e_ (N (n)) + D_e_ (2)* | | | | *AUC_g,o,k,n,a,z,mul_* ← *test (train (Net_g,o,k,n,a,z,mul_))* | *AUC_g,o,k,n,a,z,mul,max_ = max (AUC_g,o,k,n,a,z,mul_)|_for all o,k,n,a,mul_* | *if g > 1* | | *if AUC_g,o,k,n,a,z,mul,max_–AUC_g-1,o,k,n,a,z,mul,max_ ≤ t_r_* | | | *if AUC_g,o,k,n,a,z,mul,max_ > AUC_g-1,o,k,n,a,z,mul,max_* | | | | *BestNet = Net_g,o,k,n,a,z,mul_|AUC_g,o,k,n,a,z,mul,max_* | | | *else* | | | | *BestNet = Net_g−1,o,k,n,a,z,mul_|AUC_g-1,o,k,n,a,z,mul,max_* | | | *break* | | *else* | | | *BestNet = Net_g,o,k,n,a,z,mul_|AUC_g,o,k,n,a,z,mul,max_* | | | *return BestNet*	*HOSA-LSTM (Data, Gr_max_, Nh_max_, Nh_start_, Nh_step_, T_max_, T_start_, T_step_, t_r_)* *Gr* = [1, 2, …, *Gr_max_]* *T = [T_start_, T_start_ + T_step_, …, T_max_]* *Nh = [Nh_start_, Nh_start_ + Nh_step_, …, Nh_max_]* *L =* [LSTM, BLSTM] | for *t* = 1 to length *(T)* | | for *n =* 1 to length *(Nh)* | | | for *g =* 1 to length *(Gr)* | | | | for *l =* 1 to length *(L)* | | | | | *Layer = L (l)* | | | | | for *m* = 1 to 4 | | | | | | *Net_0,l,t,n,0,m_* ← *I_p_ (Data, T (t))* | | | | | | for *z* = 1 to *g* | | | | | | | *Net_z,l,t,n,0,m_* ← *Net_z-1,l,t,n,0,m_ + Layer (Nh (n))* | | | | | | if *m* == 1 | | | | | | | *N_prev_ = floor (Nh (n)*/2 + 1/2) | | | | | | | *Net_g,l,t,n,1,m_* ← *Net_g,l,t,n,0,m_ + D_e_ (N_prev_) + D_e_ (2)* | | | | | | else | | | | | | | if *m* == 2 | | | | | | | | *N_prev_ = Nh (n)* | | | | | | | | *Net_g,l,t,n,1,m_* ← *Net_g,l,t,n,0,m_ + D_e_ (N_prev_) + D_e_ (2)* | | | | | | | else | | | | | | | | if *m* == 3 | | | | | | | | | *N_prev_ = Nh (n) × 2* | | | | | | | | | *Net_g,l,t,n,1,m_* ← *Net_g,l,t,n,0,m_ + D_e_ (N_prev_) + D_e_ (2)* | | | | | | | | else | | | | | | | | | *Net_g,l,t,n,1,m_* ← *Net_g,l,t,n,0,m_ + D_e_ (2)* | | | | | | *AUC_g,l,t,n,m_* ← *test (train (Net_g,l,t,n,1,m_))* | | | *AUC_g,l,t,n,m,max_ = max (AUC_g,l,t,n,m_)|_for all l,m_* | | | if *g* > 1 | | | | if *AUC_g,l,t,n,m,max_–AUC_g-1,l,t,n,m,max_ ≤ t_r_* | | | | | if *AUC_g,l,t,n,m,max_ > AUC_g-1,l,t,n,m,max_* | | | | | | *BestNet_t,n_ = Net_g,l,t,n,1,m_|AUC_g,l,t,n,m,max_* | | | | | else | | | | | | *BestNet_t,n_ = Net_g-1,l,t,n,1,m_|AUC_g-1,l,t,n,m,max_* | | | | | break | | | | else | | | | | *BestNet_t,n_ = Net_g-1,l,t,n,1,m_|AUC_g-1,l,t,n,m,max_* | | | | return *BestNet*_*t*=1:*length (T), n*=1:*length(Nh)*_

**Table 3:** Performance of the developed models (mean ± standard deviation (*p*-value)) estimated using LOO.

Estimation	Metric	FFNN	1D-CNN	AFC LSTM	Features Fed LSTM
A phase	Acc (%) Sen (%) Spe (%) AUC	71.13 ± 14.77 72.58 ± 14.45 70.60 ± 18.44 0.801 ± 0.069	80.33 ± 3.55 (0.001 *) 75.45 ± 11.22 (0.948) 81.74 ± 2.94 (<0.001 *) 0.866 ± 0.050 (0.078)	80.72 ± 6.11 (0.004 *) 66.88 ± 9.57 (0.198) 83.19 ± 5.40 (0.018 *) 0.825 ± 0.068 (<0.001 *)	82.96 ± 5.54 (<0.001 *) 76.53 ± 11.24 (0.098) 83.36 ± 7.75 (<0.001 *) 0.882 ± 0.042 (<0.001 *)
NREM	Acc (%) Sen (%) Spe (%) AUC	73.53 ± 8.43 68.81 ± 11.96 85.40 ± 10.31 0.829 ± 0.043	78.17 ± 7.77 (<0.001 *) 81.46 ± 12.32 (<0.001 *) 71.77 ± 19.15 (1.000) 0.880 ± 0.062 (<0.001 *)	84.83 ± 5.54 (0.004 *) 89.79 ± 6.62 (<0.001 *) 73.57 ± 13.14 (1.000) 0.913 ± 0.056 (<0.001 *)	87.81 ± 6.18 (<0.001 *) 88.24 ± 7.88 (<0.001 *) 86.87 ± 11.04 (0.271) 0.945 ± 0.036 (<0.001 *)
CAP cycles	Acc (%) Sen (%) Spe (%)	70.00 ± 12.49 48.39 ± 19.36 83.27 ± 10.90	72.63 ± 10.98 (<0.001 *) 52.68 ± 20.92 (<0.001 *) 84.59 ± 7.49 (0.948)	77.69 ± 6.64 (0.003 *) 72.51 ± 13.63 (0.067) 80.53 ± 8.22 (0.384)	78.91 ± 5.17 (<0.001 *) 69.67 ± 15.63 (<0.001 *) 82.28 ± 9.91 (0.779)
CAP rate	Percentage error(%)	39.86 ± 31.79	31.77 ± 33.29	17.19 ± 14.71	21.80 ± 14.96

* Indicates a statistically significantly result.

**Table 4:** Comparative analysis between the results from the methods proposed in the state-of-the-art and the proposed methods for the A phase classification.

Work	Number of Examined Subjects	Method	Acc (%)	Sen (%)	Spe (%)	Average * (%)
[29]	13	EEG signal fed a DSAE	67	55	69	64
[24]	8	Differential variance classified by a threshold	72	52	76	67
[16]	15	EEG signal fed an LSTM	76	75	77	76
[28]	13	Auto-covariance, Shannon entropy, TEO, and frequency domain features fed an FFNN	79	76	80	78
[22]	12	Moving averages classified by a threshold	81	85	78	81
[23]	6	Similarity analysis with reference windows	81	76	81	79
[20]	4	Band descriptors, Hjorth descriptors, and differential variance classified by an FFNN	82	76	83	80
[19]	15	Entropy-based features, TEO, differential variance, and frequency-based features fed an LSTM	83	76	84	81
[21]	10	Band descriptors classified by a threshold	84	-	-	-
[25]	4	Band descriptors, Hjorth descriptors, and differential variance classified by an SVM	84	74	86	81
[26]	8	Band descriptors, Hjorth descriptors, and differential variance classified by an LDA	85	73	87	82
[27]	16	Variable windows fed to three discriminant functions	86	67	90	81
Proposed work– 1D-CNN	19	Overlapping windows fed a 1D-CNN	80	76	82	79
Proposed work–AFC LSTM	19	Pre-processed EEG signal fed an LSTM	81	67	83	77
Proposed work–FFNN	19	Amplitude, frequency, and amplitude-frequency-based features fed an FFNN	71	73	70	71
Proposed work– feature-based LSTM	19	Amplitude, frequency, and amplitude-frequency-based features fed an LSTM	83	77	83	81

* Average assessed by (Acc+Sen+Spe)/3.

## Data Availability

All data employed in this work is freely available at https://doi.org/10.13026/C2VC79 (accessed on 16 March 2022).

## Appendix A

**Table A1:** Performance of the 1D-CNN for the A phase, NREM and CAP assessments using LOO.

	A Phase				NREM				CAP		
Subject	Acc (%)	Sen (%)	Spe (%)	AUC	Acc (%)	Sen (%)	Spe (%)	AUC	Acc (%)	Sen (%)	Spe (%)
1	82.37	84.61	82.07	0.911	86.11	92.52	66.13	0.927	73.40	70.49	74.98
2	77.16	75.76	77.32	0.842	79.04	88.79	55.54	0.880	70.82	64.21	73.08
3	79.87	73.94	80.37	0.851	80.01	83.24	73.22	0.886	73.34	40.14	83.77
4	79.41	85.77	78.91	0.900	85.46	87.57	82.63	0.933	77.46	76.08	77.82
5	82.25	84.92	81.93	0.912	85.57	82.36	94.79	0.926	80.09	77.11	81.87
6	83.53	81.49	83.86	0.900	80.12	71.98	98.22	0.937	75.29	49.47	91.34
7	80.45	93.37	79.17	0.938	80.91	75.42	92.76	0.922	72.93	47.87	83.42
8	76.58	83.62	75.72	0.872	81.64	86.83	70.80	0.896	72.41	64.33	76.00
9	84.16	83.60	84.20	0.910	70.77	62.87	86.52	0.884	81.18	37.26	92.29
10	79.44	57.92	82.01	0.818	77.72	74.70	83.84	0.864	77.37	24.44	90.11
11	80.62	72.01	81.56	0.858	68.94	68.94	68.93	0.769	80.20	48.96	91.76
12	84.08	82.75	84.27	0.903	85.33	96.74	67.36	0.955	86.41	82.12	88.53
13	85.77	76.31	87.06	0.889	76.14	96.23	35.76	0.903	73.05	66.55	75.33
14	83.56	87.82	82.94	0.924	85.23	86.48	81.31	0.920	78.65	74.28	80.94
15	84.44	73.36	86.24	0.872	74.71	97.03	17.20	0.842	81.10	70.71	88.22
16	77.57	60.76	79.08	0.782	74.63	83.08	63.25	0.823	76.14	22.09	94.02
17	78.07	61.17	84.09	0.824	73.71	73.54	74.47	0.822	44.42	10.07	99.59
18	72.37	58.75	78.74	0.758	85.63	90.96	72.43	0.915	61.91	49.21	76.12
19	74.57	55.71	83.52	0.789	53.62	48.47	78.53	0.707	43.90	25.59	87.96
Mean	80.33	75.45	81.74	0.866	78.17	81.46	71.77	0.880	72.63	52.68	84.59
Standard deviation	3.55	11.22	2.94	0.050	7.77	12.32	19.15	0.062	10.98	20.92	7.49

**Table A2:** Performance of the AFC LSTM for the A phase, NREM and CAP assessments using LOO.

	A Phase				NREM				CAP		
Subject	Acc (%)	Sen (%)	Spe (%)	AUC	Acc (%)	Sen (%)	Spe (%)	AUC	Acc (%)	Sen (%)	Spe (%)
1	79.96	75.25	80.60	0.852	85.79	92.52	64.79	0.924	73.61	79.65	70.31
2	83.00	73.15	84.44	0.863	88.22	94.31	78.61	0.957	83.53	84.52	83.04
3	83.17	72.39	84.74	0.862	88.52	94.11	79.71	0.957	83.16	84.55	82.48
4	82.41	72.01	83.93	0.853	88.24	93.73	79.58	0.953	82.57	81.09	83.31
5	85.40	61.16	89.08	0.856	87.16	88.06	84.30	0.927	81.73	67.49	90.60
6	83.07	66.37	85.72	0.830	87.81	91.27	80.11	0.941	80.61	77.67	82.44
7	81.68	84.94	81.35	0.897	89.57	92.76	82.69	0.950	76.35	75.98	76.50
8	82.37	72.00	83.89	0.854	88.30	93.13	80.69	0.953	82.73	82.91	82.64
9	91.14	61.44	93.30	0.877	91.31	91.01	91.91	0.949	83.84	48.36	92.82
10	82.41	73.19	83.76	0.859	88.56	93.99	79.99	0.958	82.43	83.81	81.75
11	84.03	55.87	87.10	0.812	80.02	76.26	85.33	0.881	81.90	59.89	90.05
12	83.91	78.84	84.65	0.888	89.56	94.00	82.56	0.960	84.96	87.57	83.67
13	80.39	73.14	81.38	0.843	82.85	93.42	61.59	0.899	71.61	80.68	68.42
14	85.64	74.86	87.21	0.885	90.42	96.12	72.57	0.958	82.62	81.89	83.01
15	77.31	61.99	79.79	0.785	81.46	95.80	44.47	0.905	73.16	72.59	73.55
16	78.81	52.75	81.16	0.749	72.46	81.56	60.20	0.825	73.07	45.64	82.14
17	77.76	52.90	86.63	0.775	73.54	71.19	83.55	0.841	60.86	41.45	92.03
18	62.02	51.49	66.94	0.628	78.41	86.46	58.43	0.839	67.94	71.68	63.74
19	69.23	57.01	75.04	0.703	79.60	86.38	46.75	0.763	69.43	70.16	67.65
Mean	80.72	66.88	83.19	0.825	84.83	89.79	73.57	0.913	77.69	72.51	80.53
Standard deviation	6.11	9.57	5.40	0.068	5.54	6.62	13.14	0.056	6.64	13.63	8.22

**Table A3:** Performance of the FFNN for the A phase, NREM and CAP assessments using LOO.

	A Phase				NREM				CAP		
Subject	Acc (%)	Sen (%)	Spe (%)	AUC	Acc (%)	Sen (%)	Spe (%)	AUC	Acc (%)	Sen (%)	Spe (%)
1	81.96	76.33	82.72	0.866	79.01	77.85	82.63	0.842	75.33	51.72	88.20
2	45.50	91.34	40.19	0.757	72.28	67.82	83.01	0.802	67.87	65.70	68.61
3	76.09	59.01	77.53	0.750	70.74	62.91	87.15	0.799	69.35	27.51	82.49
4	64.14	85.15	62.47	0.813	81.54	73.37	92.49	0.877	80.29	68.81	83.22
5	85.23	76.47	86.56	0.886	74.47	68.42	93.61	0.845	81.46	58.82	95.55
6	81.56	73.53	82.83	0.853	74.58	66.45	92.64	0.849	76.49	50.60	92.57
7	80.98	84.16	80.67	0.890	76.20	67.39	95.16	0.871	73.32	47.36	84.18
8	37.51	92.57	30.75	0.752	77.92	77.00	79.85	0.846	62.00	65.15	60.58
9	91.57	51.69	94.47	0.887	67.29	52.40	96.90	0.846	82.17	24.41	96.78
10	79.35	46.64	83.25	0.768	80.56	76.09	89.61	0.877	74.29	40.10	82.51
11	82.56	59.90	85.03	0.820	75.35	75.52	75.10	0.811	79.77	47.98	91.53
12	78.32	78.50	78.29	0.846	82.55	83.13	81.64	0.878	82.94	67.01	90.81
13	76.16	71.82	76.75	0.801	82.56	84.85	77.95	0.867	69.84	62.84	72.30
14	85.21	69.26	87.54	0.886	73.76	73.37	74.99	0.820	76.36	45.76	92.35
15	49.03	92.14	42.07	0.795	76.26	85.43	52.64	0.769	71.24	85.84	61.24
16	65.68	74.02	64.93	0.753	77.06	67.15	90.37	0.832	74.55	53.99	81.35
17	74.43	54.69	81.47	0.762	54.37	44.46	96.32	0.788	41.45	5.75	98.68
18	59.01	52.42	62.09	0.614	70.78	62.19	92.03	0.832	52.15	27.95	79.15
19	57.16	89.38	41.89	0.727	49.70	41.64	88.56	0.702	39.15	22.14	80.00
Mean	71.13	72.58	70.60	0.801	73.53	68.81	85.40	0.829	70.00	48.39	83.27
Standard deviation	14.77	14.45	18.44	0.069	8.43	11.96	10.31	0.043	12.49	19.36	10.90

**Table A4:** Performance of the LSTM fed with features for the A phase, NREM and CAP assessments using LOO.

	A Phase				NREM				CAP		
Subject	Acc (%)	Sen (%)	Spe (%)	AUC	Acc (%)	Sen (%)	Spe (%)	AUC	Acc (%)	Sen (%)	Spe (%)
1	85.22	79.66	85.97	0.907	89.84	88.64	93.59	0.956	79.83	69.60	85.41
2	85.67	81.82	86.24	0.906	93.73	94.75	92.11	0.979	84.75	81.87	86.17
3	85.17	61.87	87.14	0.823	82.36	77.93	91.67	0.925	78.34	40.26	90.31
4	84.90	82.67	85.22	0.898	91.71	94.55	87.22	0.975	84.47	85.50	83.96
5	88.07	76.81	89.77	0.923	89.24	86.89	96.70	0.964	85.88	72.02	94.52
6	83.51	79.76	84.11	0.886	89.93	88.53	93.05	0.956	82.25	76.57	85.78
7	84.01	91.77	83.24	0.946	95.53	96.17	94.16	0.984	78.99	81.96	77.74
8	73.23	90.40	71.12	0.898	85.21	87.65	80.12	0.924	69.36	80.78	64.21
9	91.24	78.08	92.20	0.939	93.20	93.34	92.92	0.967	84.68	54.68	92.28
10	83.80	48.49	88.01	0.816	95.48	94.73	97.03	0.986	75.91	50.89	81.93
11	83.58	66.57	85.43	0.851	89.80	86.86	93.95	0.952	77.34	66.64	81.30
12	85.36	84.20	85.53	0.919	90.71	93.12	86.91	0.970	82.83	78.04	85.20
13	84.87	79.22	85.65	0.897	89.67	96.52	75.86	0.951	75.15	78.88	73.83
14	86.78	87.70	86.65	0.938	95.05	96.43	90.69	0.982	84.71	84.50	84.83
15	78.84	73.82	79.65	0.838	82.34	94.42	51.12	0.923	80.73	79.27	81.74
16	87.32	55.03	90.23	0.842	82.53	77.42	89.42	0.906	78.25	32.01	93.56
17	83.58	70.06	88.40	0.873	73.59	68.82	93.89	0.921	67.52	50.83	94.35
18	71.87	83.77	66.30	0.842	77.49	76.48	80.01	0.877	72.72	76.24	68.78
19	69.26	82.39	63.03	0.821	80.97	83.21	70.14	0.858	75.60	83.17	57.38
Mean	82.96	76.53	83.36	0.882	87.81	88.24	86.87	0.945	78.91	69.67	82.28
Standard deviation	5.54	11.24	7.75	0.042	6.18	7.88	11.04	0.036	5.17	15.63	9.91

**Table A5:** Performance of the developed methods for the CAP rate assessment using LOO.

	Model Based on the 1D-CNN		Model Based on the AFC LSTM		Model Based on the FFNN		Model Based on the LSTM Fed with Features	
Subject	CAP rate error (%)	CAP rate percentage error	CAP rate error (%)	CAP rate percentage error	CAP rate error (%)	CAP rate percentage error	CAP rate error (%)	CAP rate percentage error
1	5.94	12.64	13.60	28.94	−5.59	11.89	2.93	6.23
2	12.01	25.55	5.54	11.79	39.67	84.40	5.30	11.28
3	−1.68	3.57	6.60	14.04	8.08	17.19	−4.56	9.70
4	22.45	47.77	4.14	8.81	25.09	53.38	8.29	17.64
5	14.82	31.53	−5.60	11.91	−2.99	6.36	−4.14	8.81
6	−7.12	15.15	3.25	6.91	−4.97	10.57	4.87	10.36
7	3.50	7.45	13.39	28.49	9.91	21.09	15.82	33.66
8	7.54	16.04	6.48	13.79	34.82	74.09	29.84	63.49
9	2.16	4.60	−5.87	12.49	−9.11	19.38	−3.67	7.81
10	−4.72	10.04	7.09	15.09	11.51	24.49	8.94	19.02
11	−11.86	25.23	−0.06	0.13	−10.94	23.28	13.00	27.66
12	−5.47	11.64	8.08	17.19	−4.76	10.13	3.52	7.49
13	2.35	5.00	20.13	42.83	18.42	39.19	16.09	34.23
14	8.60	18.30	4.10	8.72	−12.40	26.38	6.39	13.60
15	−18.04	38.38	−2.98	6.34	22.13	47.09	−3.93	8.36
16	−26.28	55.91	−4.49	9.55	20.77	44.19	−17.54	37.32
17	−67.26	143.11	−29.35	62.45	−65.13	138.57	−17.61	37.47
18	−22.98	48.89	0.71	1.51	−21.75	46.28	17.29	36.79
19	−38.97	82.91	−12.07	25.68	−27.92	59.40	10.94	23.28
Mean	-	31.77	-	17.19	-	39.86	-	21.80
Median	−1.68	18.30	4.10	12.49	−2.99	26.38	5.30	17.64
Standard deviation	-	33.29	-	14.71	-	31.79	-	14.96

## References

[1] Berry R., Brooks R., Gamaldo C., Harding S., Lloyd R., Marcus C., Vaughn B. (2017). The AASM Manual for the Scoring of Sleep and Associated Events: Rules, Terminology and Technical Specifications.

[2] Kubicki S., Herrmann W. (1996). The Future of Computer-Assisted Investigation of the Polysomnogram: Sleep Microstructure. J. Clin. Neurophysiol..

[3] Terzano M., Parrino L. (2000). Origin and Significance of the Cyclic Alternating Pattern (CAP). Sleep Med. Rev..

[4] Terzano M., Parrino L., Sherieri A., Chervin R., Chokroverty S., Guilleminault C., Hirshkowitz M., Mahowald M., Moldofsky H., Rosa A. (2001). Atlas, Rules, and Recording Techniques for the Scoring of Cyclic Alternating Pattern (CAP) in Human Sleep. Sleep Med..

[5] Halász P., Terzano M., Parrino L., Bódizs R. (2004). The Nature of Arousal in Sleep. J. Sleep Res..

[6] Parrino L., Ferri R., Bruni O., Terzano M. (2012). Cyclic Alternating Pattern (CAP): The Marker of Sleep Instability. Sleep Med. Rev..

[7] Terzano M., Parrino L. (1993). Clinical Applications of Cyclic Alternating Pattern. Physiol. Behav..

[8] Parrino L., Milioli G., Melpignano A., Trippi I. (2016). The Cyclic Alternating Pattern and the Brain-Body-Coupling during Sleep. Epileptologie.

[9] Terzano M., Parrino L., Boselli M., Spaggiari M., Di Giovanni G. (1996). Polysomnographic Analysis of Arousal Responses in Obstructive Sleep Apnea Syndrome by Means of the Cyclic Alternating Pattern. J. Clin. Neurophysiol..

[10] Largo R., Lopes M., Spruyt K., Guilleminault C., Wang Y., Rosa A. (2019). Visual and Automatic Classification of the Cyclic Alternating Pattern in Electroencephalography during Sleep. Braz. J. Med. Biol. Res..

[11] Rosa A., Alves G., Brito M., Lopes M., Tufik S. (2006). Visual and Automatic Cyclic Alternating Pattern (CAP) Scoring: Inter-Rater Reliability Study. Arq. Neuro-Psiquiatr..

[12] Mendez M., Alba A., Chouvarda I., Milioli G., Grassi A., Terzano M., Parrino L. On separability of A-phases during the cyclic alternating pattern. Proceedings of the 2014 36th Annual International Conference of the IEEE Engineering in Medicine and Biology Society.

[13] Hartmann S., Baumert M. (2019). Automatic A-Phase Detection of Cyclic Alternating Patterns in Sleep Using Dynamic Temporal Information. IEEE Trans. Neural Syst. Rehabil. Eng..

[14] Mostafa S., Mendonça F., Ravelo-García A., Juliá-Serdá G., Morgado-Dias F. (2020). Multi-Objective Hyperparameter Optimization of Convolutional Neural Network for Obstructive Sleep Apnea Detection. IEEE Access.

[15] Kiranyaz S., Avci O., Abdeljaber O., Ince T., Gabbouj M., Inman D. (2021). 1D Convolutional Neural Networks and Applications: A Survey. Mech. Syst. Signal Process..

[16] Mendonça F., Mostafa S., Morgado-Dias F., Ravelo-García A. (2019). A Portable Wireless Device for Cyclic Alternating Pattern Estimation from an EEG Monopolar Derivation. Entropy.

[17] Zhao J., Obonyo E. (2020). Convolutional Long Short-Term Memory Model for Recognizing Construction Workers’ Postures from Wearable Inertial Measurement Units. Adv. Eng. Inform..

[18] Mendonça F., Mostafa S., Morgado-Dias F., Ravelo-García A. (2020). On the Use of Patterns Obtained from LSTM and Feature-Based Methods for Time Series Analysis: Application in Automatic Classification of the CAP A Phase Subtypes. J. Neural Eng..

[19] Hartmann S., Baumert M. Improved A-Phase Detection of Cyclic Alternating Pattern Using Deep Learning. Proceedings of the 2019 41st Annual International Conference of the IEEE Engineering in Medicine and Biology Society (EMBC).

[20] Mariani S., Bianchi A., Manfredini E., Rosso V., Mendez M., Parrino L., Matteucci M., Grassi A., Cerutti S., Terzano M. Automatic Detection of A Phases of the Cyclic Alternating Pattern during Sleep. Proceedings of the 2010 Annual International Conference of the IEEE Engineering in Medicine and Biology.

[21] Barcaro U., Bonanni E., Maestri M., Murri L., Parrino L., Terzano M. (2004). A General Automatic Method for the Analysis of NREM Sleep Microstructure. Sleep Med..

[22] Largo R., Munteanu C., Rosa A. CAP Event Detection by Wavelets and GA Tuning. Proceedings of the IEEE International Workshop on Intelligent Signal Processing.

[23] Niknazar H., Seifpour S., Mikaili M., Nasrabadi A., Banaraki A. A Novel Method to Detect the A Phases of Cyclic Alternating Pattern (CAP) Using Similarity Index. Proceedings of the 2015 23rd Iranian Conference on Electrical Engineering.

[24] Mariani S., Manfredini E., Rosso V., Mendez M., Bianchi A., Matteucci M., Terzano M., Cerutti S., Parrino L. (2011). Characterization of A Phases during the Cyclic Alternating Pattern of Sleep. Clin. Neurophysiol..

[25] Mariani S., Grassi A., Mendez M., Parrino L., Terzano M., Bianchi A. Automatic Detection of CAP on Central and Fronto-Central EEG Leads via Support Vector Machines. Proceedings of the 33rd Annual International Conference of the IEEE Engineering in Medicine and Biology Society.

[26] Mariani S., Manfredini E., Rosso V., Grassi A., Mendez M., Alba A., Matteucci M., Parrino L., Terzano M., Cerutti S. (2012). Efficient Automatic Classifiers for the Detection of A Phases of the Cyclic Alternating Pattern in Sleep. Med. Biol. Eng. Comput..

[27] Mariani S., Grassi A., Mendez M., Milioli G., Parrino L., Terzano M., Bianchi A. (2013). EEG Segmentation for Improving Automatic CAP Detection. Clin. Neurophysiol..

[28] Mendonça F., Fred A., Mostafa S., Morgado-Dias F., Ravelo-García A. (2018). Automatic Detection of Cyclic Alternating Pattern. Neural Comput. Appl..

[29] Mostafa S., Mendonça F., Ravelo-García A., Morgado-Dias F. Combination of Deep and Shallow Networks for Cyclic Alternating Patterns Detection. Proceedings of the 2018 13th APCA International Conference on Automatic Control and Soft Computing (CONTROLO).

[30] Goldberger A., Amaral L., Glass L., Hausdorff M., Ivanov P., Mark R., Mietus J., Moody G., Peng C., Stanley H. (2000). PhysioBank, PhysioToolkit, and PhysioNet: Components of a New Research. Circulation.

[31] Digital Signal Processing Committee I. (1979). Programs for Digital Signal Processing.

[32] Phillips C., Parr J., Riskin E. (2013). Signals, Systems, and Transforms.

[33] Muralidharan K. (2010). A Note on Transformation, Standardization and Normalization. IUP J. Oper. Manag..

[34] Urigüen J., Zapirain B. (2015). EEG Artifact Removal—State-of-the-Art and Guidelines. J. Neural Eng..

[35] Ortigueira M. (2005). Processamento Digital de Sinais.

[36] Peng H., Long F., Ding C. (2005). Feature Selection Based on Mutual Information Criteria of Max-Dependency, Max-Relevance, and Minredundancy. IEEE Trans. Pattern Anal. Mach. Intell..

[37] Goodfellow I., Bengio Y., Courville A. (2016). Deep Learning.

[38] Kiranyaz S., Ince T., Hamila R., Gabbouj M. Convolutional Neural Networks for Patient-Specific ECG Classification. Proceedings of the 2015 37th Annual International Conference of the IEEE Engineering in Medicine and Biology Society (EMBC).

[39] Kiranyaz S., Ince T., Gabbouj M. (2016). Real-Time Patient-Specific ECG Classification by 1-D Convolutional Neural Networks. IEEE Trans. Biomed. Eng..

[40] Mendonça F., Mostafa S., Morgado-Dias F., Juliá-Serdá G., Ravelo-García A. (2020). A Method for Sleep Quality Analysis Based on CNN Ensemble With Implementation in a Portable Wireless Device. IEEE Access.

[41] Hochreiter S., Schmidhuber J. (1997). Long Short-Term Memory. Neural Comput..

[42] Sackett D., Haynes R., Guyatt G., Tugwell P. (1991). Clinical Epidemiology: A Basic Science for Clinical Medicine.

[43] Fawcett T. (2006). An Introduction to ROC Analysis. Pattern Recognit. Lett..

[44] Kingma D., Ba J. (2015). Adam: A Method for Stochastic Optimization. arXiv.

[45] Mostafa S., Baptista D., Ravelo-García A., Juliá-Serdá G., Morgado-Dias F. (2020). Greedy Based Convolutional Neural Network Optimization for Detecting Apnea. Comput. Methods Programs Biomed..

[46] Yamashita R., Nishio M., Do R., Togashi K. (2018). Convolutional Neural Networks: An Overview and Application in Radiology. Insights Imaging.

[47] Guidici D., Clark M. (2017). One-Dimensional Convolutional Neural Network Land-Cover Classification of Multi-Seasonal Hyperspectral Imagery in the San Francisco Bay Area, California. Remote Sens..

[48] Ng W., Minasny B., Montazerolghaem M., Padarian J., Ferguson R., Bailey S., McBratney A. (2019). Convolutional Neural Network for Simultaneous Prediction of Several Soil Properties Using Visible/near-Infrared, Mid-Infrared, and Their Combined Spectra. Geoderma.

[49] Thai-Nghe N., Gantner Z., Schmidt-Thieme L. Cost-Sensitive Learning Methods for Imbalanced Data. Proceedings of the 2010 International Joint Conference on Neural Networks (IJCNN).

[50] Kohavi R. (1995). A Study of Cross-Validation and Bootstrap for Accuracy Estimation and Model Selection.

[51] Yadav A., Jha C., Sharan A. (2020). Optimizing LSTM for Time Series Prediction in Indian Stock Market. Procedia Comput. Sci..

[52] Shen H., Ran F., Xu M., Guez A., Li A., Guo A. (2020). An Automatic Sleep Stage Classification Algorithm Using Improved Model Based Essence Features. Sensors.

